# How the Stringency of the COVID-19 Restrictions Influences Motivation for Adherence and Well-Being: The Critical Role of Proportionality

**DOI:** 10.34172/ijhpm.2023.8021

**Published:** 2023-10-10

**Authors:** Joachim Waterschoot, Sofie Morbée, Omer Van den Bergh, Vincent Yzerbyt, Eveline Raemdonck, Marie Brisbois, Mathias Schmitz, Olivier Klein, Olivier Luminet, Pascaline Van Oost, Maarten Vansteenkiste

**Affiliations:** ^1^Department of Developmental, Personality and Social Psychology, Ghent University, Ghent, Belgium.; ^2^Health Psychology, Faculty of Psychology and Educational Sciences, University of Leuven, Leuven, Belgium.; ^3^Institute for Research in the Psychological Sciences, Université Catholique de Louvain, Louvain-la-Neuve, Belgium.; ^4^Faculty of Psychological Sciences and Education, Université libre de Bruxelles, Bruxelles, Belgium.; ^5^Fund for Scientific Research (FRS-FNRS), Brussels, Belgium.

**Keywords:** Epidemiology, Policy, Proportionality, Motivation, Risk Perception, Well-Being

## Abstract

**Background:** The stringency of the measures taken by governments to combat the COVID-19 pandemic varied considerably across countries and time. In the present study, we examined how the proportionality to the epidemiological situation is related to citizens’behavior, motivation and mental health.

**Methods:** Across 421 days between March 2020 and March 2022, 273,722 Belgian participants (*M_age_* = 49.47; 63.9% female; 33% single) completed an online questionnaire. Multiple linear mixed regression modeling was used to examine the interaction between the epidemiological situation, as indicated by the actual hospitalization numbers, and the stringency index to predict day-to-day variation in the variables of interest.

**Results:** Systematic evidence emerged showing that disproportional situations, as opposed to proportional situations, were associated with a clear pattern of maladaptive outcomes. Specifically, when either strict or lenient measures were disproportional in relation to the epidemiological situation, people reported lower autonomous motivation, more controlled motivation and amotivation, less adherence to sanitary rules, higher perceived risk of infection, lower need satisfaction, and higher anxiety and depressive symptoms. Perceived risk severity especially covaried with the stringency of the measures. At the absolute level, citizens reported the highest need satisfaction and mental health during days with proportional lenient measures.

**Conclusion:** Stringent measures are not per se demotivating or compromising of people’s well-being, nor are lenient measures as such motivating or enhancing well-being. Only proportional measures, that is, measures with a level of stringency that is aligned with the actual epidemiological situation, are associated with the greatest motivational, behavioral, and mental health benefits.

## Background

**Key Messages**

**Implications for policy makers**

- The current findings provide a unique and informative insight into how the role of (dis)proportional measures affects different aspects of psychological functioning in the population.
- The implementation of stricter measures is not per se demotivating or psychologically damaging if it is proportional and legitimate to what is required.
- Milder measures are not per se motivating because they may be perceived as lax and insufficient when a threat requires a political response.
- The monitoring of psychological aspects of the population over time is critical in order to closely observe how the population’s perception is affected by even objective parameters and implementations.
- During uncertain times such as the COVID-19 crisis, instruments such as a Corona Barometer should be implemented to provide a number of psychological advantages, such as a greater sense of control and predictability.

**Implications for the public**

During the COVID-19 crisis, people’s motivation and mental health were affected by the epidemiological situation (ie, the number of hospitalizations) and the political situation (ie, the implementation of measures). Although previous work has shown that stricter measures led to lower levels of well-being,^1^ we tested the proportionality hypothesis and showed that stricter measures led to lower levels of mental health and motivation only when they were not proportional to the epidemiological situation. Conversely, in times of high hospitalization rates, lenient policies led to higher perceived change in infection and more amotivation. The most ideal pattern was found when both political and epidemiological situations were proportional. These results show that although a proportional situation was required at the macro level, people were willing to control the pandemic without psychological costs. Therefore, as long as the situation requires clear and transparent rules, strict measures could be required.

The COVID-19 crisis posed an extreme threat to human health. An infection with the SARS-CoV-2 virus could not only come with a variety of disabling symptoms (eg, coughing, difficulty breathing, headache, and fatigue) but could also result in hospitalization in case of severe symptoms. To illustrate, in Belgium, where the present long-term study took place, more than 146 000 people were hospitalized throughout the pandemic, with 1465 new people requiring daily intensive care at the peak of the second wave.^2^ To prevent a collapse of the healthcare system and to save lives, governments around the world took a range of restrictive measures that varied in severity and duration. In some cases, authorities enforced a lockdown, travel bans and a restricted mobility perimeter, allowing citizens only to move around within a narrow radius of their homes. These measures proved effective in reducing the spread of the virus.^3,4^

###  The Interplay Between the Stringency of the Measures and the Hospitalization Numbers 

 Across countries and time, hospitalization numbers, a reliable index of the actual epidemiological situation, and the stringency of the implemented measures did not always go hand in hand.^5^ With comparable hospitalization numbers on average, some countries (eg, China) imposed more severe restrictions than others (eg, Sweden). Similarly, countries also differed in how quickly the measures were adapted to changes in the epidemiological situation.^6^ In addition, some governments relied on economic indicators (eg, economic growth^7^), while others had predetermined epidemiological markers to adapt the stringency of the measures.^8^ Overall, countries that lacked a coherent policy proved less efficient in facing the pandemic, whereas countries that monitored infections closely and implemented clear and consistent measures using transparent and efficient communication strategies, were far more effective in combatting the crisis.^9^

 Most countries were not adequately prepared to face the pandemic and needed to learn along the road to find a balance between the stringency of the measures that was needed to control the epidemiological situation and the collateral damage at the economic, social, and psychological levels.^10^ Indeed, imposed behavioral restrictions were often fiercely criticized for producing economic loss,^11^ and stimulated conspiracy thinking,^12^ street protests^13^ and societal debate questioning their necessity.^14^ Often people seemed to lose their motivation to adhere to the measures,^15^ even defying the measures altogether, thereby endangering the health of others. Apparently, psychological costs emerged because strict measures violated people’s basic needs for autonomy, competence, and relatedness,^16,17^ thereby undermining people’s mental health as evidenced through enhanced feelings of insecurity,^18^ decreased vitality,^19^ and reduced life satisfaction.^20^

 However, lenient measures were not necessarily better. Indeed, the slow introduction of strict measures in times of rising hospitalizations was criticized because governments came across as too passive, insufficiently reliable or incompetent to protect the populations’ safety and mental health.^12^ Lenient measures may also be an external signal to people that the situation is not quite serious, which would contradict their internal signal of higher risk perception. Interestingly, too lenient measures stimulated a sizeable proportion of the population to spontaneously engage in health-protective behavior such as avoiding social contacts^21^ and public transportation.^22^ These spontaneous self-restrictions apparently reduced feelings of uncertainty and ambiguity that were provoked by inadequate governmental interventions to contain the (perceived) health threat.^23^

###  Present Study

 Although prior studies have mainly focused on relating psychological variables to either the epidemiological situation^1^ or to stringency of the measures,^24^ the above description suggests that their impact is not one-sidedly positive or negative. What may be especially critical is the interaction between both, with the impact of the stringency of behavioral measures varying as a function of the epidemiological threat. In a nutshell, the proportionality between the measures and the actual health situation is of paramount importance with higher fit translating into a better psychological response of the population. Collecting data across the entire pandemic in Belgium, the current study seeks, as far as we know for the first time, to examine the interplay between the actual epidemiological situation (in terms of the hospitalization load) and the stringency of the measures (in terms of the stringency index^5^) in predicting a wide range of behavioral, motivational, and mental health outcomes.

 Specifically, we focus on people’s self-reported adherence to the measures and their motivation for doing so within the framework of the Self-Determination Theory.^16^ In addition to amotivation (ie, denoting a low level of motivation), two qualitatively different types of motivation are discerned. Autonomous or high-quality motivation reflects the full endorsement of measures out of personal value and conviction, whereas controlled or poor-quality motivation represents pressured form of motivation (eg, to avoid sanctions). Higher mental health is evidenced when the basic psychological needs for autonomy (perceived authenticity and psychological freedom), competence (perceived mastery and efficacy in the things one do) and relatedness (feeling warm relationships with others) are fulfilled and was assessed through of the presence of vitality and life satisfaction and absence of symptoms of depression and anxiety.^1^

 The herein proposed *proportionality hypothesis* specifically aimed to shed light on the interplay between these stringency and epidemiological threat.^25,26^ Specifically, we hypothesized that the level of behavioral adherence, motivation, risk perception, and mental health of the population depends on the proportionality (or fit) between the stringency of the measures and the actual epidemiological threat. We tested our proportionality hypothesis from two angles. First, we reasoned that a higher daily hospitalization load would predict a less adaptive pattern of motivation and well-being outcomes (eg, vitality, depression symptoms) when a more lenient, instead of a stricter, set of measures prevails. For instance, stricter measures would buffer against the higher anxiety experienced on days with a high number of hospitalizations. Also, stricter measures would not necessarily be experienced as autonomy-constraining because, if they come across as appropriate in the given circumstances, they may foster endorsement of and commitment to the measures. Conversely, the absence of strict measures on days with high hospitalization load would erode people’s motivation to adhere to the measures, possibly because it may elicit a sense of helplessness and lack of control. A second way to examine the interplay between hospitalization load and stringency of the measures is to zoom in on those days with low hospitalizations rates. If the measures in place are still strict on these days, they may be experienced as frustrating basic needs for autonomy, competence, and relatedness and undermining people’s mental health. Conversely, lenient measures in such situation will afford greater opportunities for basic need satisfaction and improved mental health.^26^

## Methods

###  Participants and Procedure

 From the first day of the Belgian lockdown, we launched an online questionnaire through social media platforms and newspapers. We advertised the study as dealing with people’s experiences during lockdown. Prior to completing the questionnaire, participants signed an informed consent in which the voluntary nature of the study was emphasized. People could quit anytime without negative consequences and the collected data would be handled confidentially. Initially, we distributed the questionnaire on a daily basis but the pace of collecting data went down after 60 days.

 We collected data from March 19, 2020 until May 16, 2022 (ie, 788 days), with at least 30 participants completing the questionnaire on any given day for 421 days (ie, 53.4%). This periods contains 211 days (50%) before March 2021, the month in which the vaccination campaign for the total population started. In total, 273 722 participants (*M*_age_ = 49.47; 63.9% female; 33% single) completed the questionnaire, with an average of 644 participants per day (range: 32–6363). Participants who already had completed the questionnaire before were excluded (ie, based on their email address and an item assessing previous completions of the questionnaire). Further, 32.4% reported to have no or secondary graduation, 36.8% had a Bachelor’s degree and 30.8% had a Master’s degree. In total, 80.6% reported to have no comorbidities with 16.2% reported to have one.

###  Measures

####  Adherence

 We assessed self-reported adherence to the four most important corona measures in Belgium (ie, washing hands, wearing a mouth mask, avoiding contact with others, and maintaining physical distance) with one item each. Participants indicated on a scale ranging from 1 (“I do not adhere to it at all”) to 5 (“I totally adhere to it”) the extent to which they followed each of the four measures. Internal consistency was acceptable, with a Cronbach’s alpha of 0.84 on the between-day level and a Cronbach’s alpha of 0.81 on the between-person level.

####  Motivation

 We assessed people’s motivation to adhere to the corona safety measures with an adapted version of the Behavioral Regulation in Sport Questionnaire.^27^ After the stem “Over the past week, I adhered to these measures…,” people answered four items for autonomous motivation (eg, “…because I find it personally relevant”; α_between-days_ = 0.89, α_between-person_ = 0.81) and 4 items for controlled motivation (eg, “…because I feel compelled to do so”; α_between-days_ = 0.86, α_between-person_ = 0.82). Additionally, we conducted 4 items for amotivation assessing reasons for not adhering the measures (eg, “…because I do not believe that the current approach to the corona crisis helps solve the problem cause”; α_between-days_ = 0.86, α_between-person_ = 0.74). Respondents rated items on a 5-point scale ranging from 1 (“not at all true”) to 5 (“totally true”). Internal consistencies were acceptable on both levels.

####  Risk Perception

 We measured risk perception with four items,^28^ two of which asked participants to estimate the probability to be infected by the coronavirus in the near future (1 = “Very small” to 5 = “Very big”; α_between-days_ = 0.83, α_between-person_ = 0.79) and two items the severity of the symptoms when infected (1 = “Not at all serious” to 5 = “Very serious”; α_between-days_ = 0.81, α_between-person_ = 0.80). Participants answered both questions twice, once with respect to themselves and once with respect to the Belgian population.

####  Psychological Need Satisfaction

 Participants completed a brief version of the Basic Psychological Need Satisfaction and Need Frustration Scale (12 items).^29^ They rated items in reference to the preceding week on a 5-point scale ranging from 1 (“not at all true”) to 5 (“totally true”). Six items assessed participants’ experience of satisfaction and six others the frustration of the psychological needs for autonomy, relatedness, and competence. To reduce the number of variables, a composite score was created by subtracting the averaged need frustration score from the averaged need satisfaction. As a result, the relative index ranged from -4 to +4 with 0 denoting the tipping point between frustration (ie, negative score) and satisfaction (ie, positive score). Example items are: “I felt that my decisions reflected what I really wanted” (ie, autonomy), “I had the impression that people I spent time with disliked me” (ie, relatedness), and “I felt confident that I could do things well” (ie, competence). Internal consistencies were acceptable for autonomy (α_between_= 0.84; α_within_= 0.65), competence (α_between_= 0.78; α_within_= 0.67) and relatedness (α_between_= 0.78; α_within_= 0.64).

###  Well-Being

####  Life Satisfaction and Vitality

 To measure life satisfaction, we selected the item “In the previous week, I was satisfied with my life.” of the Satisfaction with Life Scale^30^ in line with the study of Brenning et al.^31^ We did the same for vitality based on the Subjective Vitality Scale scale (“In the previous week, I felt energized”^32^) and asked participants to report on a scale ranging from 1 (“seldom or never, less than 1 day”) to 4 (“mostly or all the time, 5 to 7 days”). We chose this single item method for the sake of practicality^33^ while losing little validity of these measures.^34^

####  Depressive and Anxiety symptoms

 We assessed depressive symptoms by means of a 6-item version of the Center for Epidemiological Studies—Depression scale (CES-D^35,36^). We measured anxiety symptoms with a 4-item version of the State Trait Anxiety Inventory (STAI^37^). We added one item from the full version of the STAI to tap into anxiety in a more direct way (ie, “I felt anxious”). The stem (ie, “During the past week”) preceded all items and participants provided their answers on a scale ranging from 1 (“seldom or never, less than 1 day”) to 4 (“mostly or all the time, 5 to 7 days”). Internal consistencies were acceptable for both depressive symptoms (α_between_= 0.76; α_within_= 0.61) and anxiety symptoms (α_between_= 0.73; α_within_= 0.59).

####  Hospitalizations

 We secured data on hospitalizations from Sciensano, the national public health institute.^2^ As this parameter comes in exponentials, we log-transformed this variable to include it in linear analyses. The hospitalization numbers relied on the same data collection protocol throughout the period covered in our study (See Figure S1, Supplementary file 1).

####  Stringency of the Measures

 To operationalize the strictness of the implemented measures, we used the Stringency index.^5^ These authors tracked the strictness of measures across the world and generated the Oxford COVID-19 Government Response Tracker (OxCGRT). OxCGRT provides a percentage representing the level of stringency of restrictions across time and relies on nine indicators: school closures, workplace closures, cancellation of public events, restrictions on public gatherings, closures of public transport, stay-at-home requirements, public information campaigns, restrictions on domestic movements, and international travel controls. The index on a given day corresponds to the mean score of the nine metrics, each ranging from 0 (the most lenient restrictions) to 100 (the most severe restrictions; See Figure S2).

###  Plan of Analysis

 All analyses were done in Rstudio.^38^ Before inspecting the associations between variables at the between-day level, we calculated the intra-class correlation (ICC). The ICC reflects how strongly observations within days are associated and thus represents the proportion of between-day variance relative to the within-day (or between-person) variance. Because our predictors (ie, hospitalizations and stringency) are at the between-day level, a sufficient amount of variance in the outcomes needs to be at the between-day level to justify the use of a multilevel approach.^39^ Next, we calculated Pearson correlations to check for the associations between variables at the between-day and within-day level.

 In the main analyses, we examined the unique and interactive contribution of hospitalization load and stringency index. As both predictors varied only between days, we used linear mixed regression with ‘days’ as the random effect, thereby allowing the model to calculate the parameters and statistics accounting for the level of dependent variance in the dataset within days (ie, between-persons). In addition, we controlled the current associations for differences between two phases of the crisis, with phase 1 (19/03/2020–28/02/2021) as the first year of the crisis with no vaccinated people and phase 2 (01/03/2021–19/03/2022) including other variants (like omicron) and a particular number of vaccinated people in the population. Both continuous predictors were centered to keep the standard deviation (SD) of the variables original for the sake of interpretability.^40^ Also, we calculated variance-inflation-factors (VIFs) to check for the level of multicollinearity, which is indicated by VIF-values higher than 4. In their output, we rely on standardized coefficients. Also, we added effect sizes by calculating the partial eta-square, because *P *values are affected by the size of the current sample. An eta-squared of 0.01 indicates a small effect, while a value between 0.02 and 0.06 indicates a moderate effect and everything higher than 0.08 indicates a large effect.^41^

 To gain a clear understanding of significant interaction effects, we show the effect of hospitalization numbers on a given outcome by the level of the stringency index. In the output, we therefore report the Johnson-Neyman interval,^42^ showing for which values of the stringency index the simple slopes are significant (See example Figure S3). For the sake of visibility, we calculated the predicted values of the model for low (-1 SD) and high (+1 SD) levels, corresponding to, respectively, 39% and 72% for the stringency of the measures and 1130 (7.03) and 4230 (8.35) (logged) daily hospitalizations.

 For these levels, we also added standardized simple slope coefficients to the figure. Finally, to obtain a clear and summarizing overview of the various findings, involving 11 outcomes, we created a bar plot with the centered predicted values of the linear mixed regression models across four situations. These four situations are identical to the four points in the interaction figures and, hence, reflect different combinations of low vs. high hospitalizations and lenient vs. strict measures. The syntax and anonymized data can be found on https://osf.io/sa498/.

## Results

###  Preliminary Analyses

**Figure 1:**
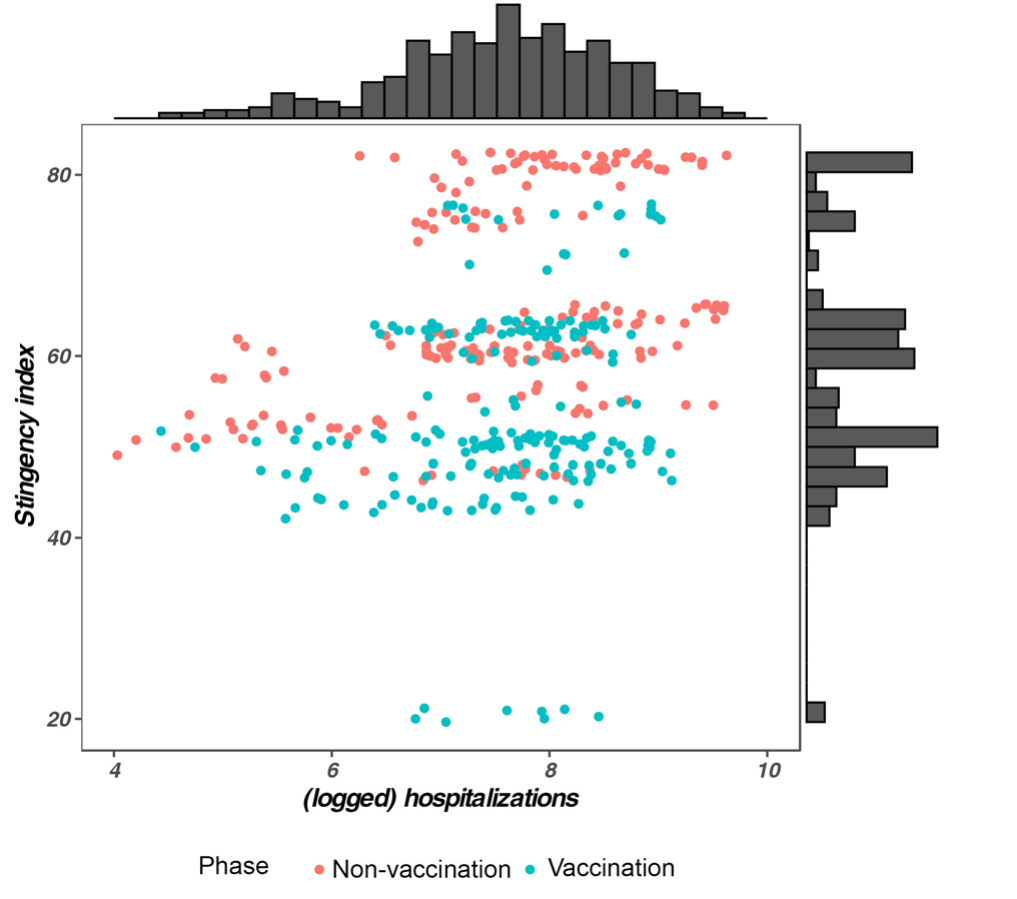
Scatterplot With Distributions Between the Stringency Index and the (Logged) Number of Hospitalizations by Phase of The Crisis. *Note. *A dot represents one day. We jittered the positions of the dots with value 1 to provide a overview of all dots without overlap.

 First, the ICC’s justified the use of a multilevel approach, with 3%-14% of the variance in the outcomes showing at the between-day level. Second, Pearson correlations can be found in Table 1, with correlations on the between-person level in the upper diagonal and those on the between-day level in the lower diagonal. The number of daily hospitalizations was positively, yet modestly, correlated with the stringency index. As Figure 1 shows, there were days during the crisis with a high number of hospitalizations and a non-stringent set of measures and vice versa, even within both crisis phases.

**Table 1:** Multilevel Pearson Correlations on the Between-Day (Lower Diagonal) and Between-Person (Upper Diagonal) Level

	**Mean**	**SD**	**ICC**	**1.**	**2.**	**3.**	**4.**	**5.**	**6.**	**7.**	**8.**	**9.**	**10.**	**11.**	**12.**	**13.**	**14.**	**15.**
Crisis-related factors																		
1. Hospitalisations	7.69	0.66	-															
2. Stringency index	55.37	16.5	-	0.23^a^														
Motivation																		
3. Adherence	3.91	1.00	0.13	0.11^b^	0.72^a^		0.63^a^	-0.26^a^	-0.49^a^	0.27^a^	0.50^a^	0.24^a^	0.04^a^	0.06^a^	0.04^a^	0.06^a^	0.01	-0.04^a^
4. Autonomous	3.45	1.17	0.13	0.12^b^	0.39^a^	0.84^a^		-0.43^a^	-0.63^a^	0.36^a^	0.61^a^	0.47^a^	0.11^a^	0.20^a^	0.11^a^	0.16^a^	-0.08^a^	-0.14^a^
5. Controlled	2.61	1.05	0.06	-0.01	0.14^c^	-0.31^a^	-0.70^a^		0.45^a^	-0.14^a^	-0.30^a^	-0.49^a^	-0.21^a^	-0.25^a^	-0.16^a^	-0.20^a^	0.20^a^	0.23^a^
6. Amotivation	2.37	0.90	0.11	0.03	-0.26^a^	-0.75^a^	-0.90^a^	0.70^a^		-0.26^a^	-0.46^a^	-0.46^a^	-0.22^a^	-0.25^a^	-0.16^a^	-0.20^a^	0.17^a^	0.23^a^
Risk perception																		
7. Perceived infection	2.98	0.82	0.07	0.31^a^	-0.02	0.01	-0.04	0.00	0.04		0.46^a^	0.13^a^	-0.05^a^	0.03^a^	-0.05^a^	-0.01^c^	0.11^a^	0.03^c^
8. Perceived severity	2.91	0.92	0.14	-0.09	0.57^a^	0.85^a^	0.73^a^	-0.11^c^	-0.69^a^	-0.09		0.29^a^	0.00	0.08^a^	0.00	0.03^c^	0.04^c^	-0.01
Psychological needs																		
9. Autonomy	0.20	1.70	0.08	-0.08	-0.43^a^	-0.03	0.45^a^	-0.88^a^	-0.46^a^	-0.07	-0.11^c^		0.45^a^	0.46^a^	0.37^a^	0.43^a^	-0.44^a^	-0.46^a^
10. Competence	1.48	1.54	0.04	-0.10^c^	-0.59^a^	-0.51^a^	-0.28^a^	-0.26^a^	0.14^c^	0.16^a^	-0.46^a^	0.50^a^		0.53^a^	0.48^a^	0.50^a^	-0.57^a^	-0.60^a^
11. Relatedness	1.72	1.46	0.03	-0.07	-0.46^a^	-0.26^a^	0.15^a^	-0.67^a^	-0.14^c^	0.04	-0.37^a^	0.86^a^	0.67^a^		0.41^a^	0.44^a^	-0.43^a^	-0.52^a^
Well-being																		
12. Vitality	2.72	1.03	0.05	-0.15^a^	-0.32^a^	-0.30^a^	-0.09	-0.33^a^	0.01	0.08	-0.41^a^	0.44^a^	0.49^a^	0.49^a^		0.65^a^	-0.64^a^	-0.60^a^
13. Life satisfaction	2.86	1.04	0.05	-0.14^c^	-0.27^a^	-0.15^a^	0.14^c^	-0.55^a^	-0.16^a^	0.05	-0.22^a^	0.63^a^	0.47^a^	0.61^a^	0.63^a^		-0.66^a^	-0.63^a^
14. Anxiety symptoms	2.13	0.80	0.09	0.19^a^	0.21^a^	0.15^a^	-0.09	0.47^a^	0.22^a^	0.11^c^	0.21^a^	-0.56^a^	-0.48^a^	-0.52^a^	-0.53^a^	-0.64^a^		0.76^a^
15. Depressive symptoms	1.7	0.68	0.05	0.10	0.32^a^	0.13^c^	-0.11^c^	0.45^a^	0.18^a^	0.02	0.24^a^	-0.53^a^	-0.60^a^	-0.53^a^	-0.28^a^	-0.46^a^	0.50^a^	

Abbreviations: ICC, intra-class correlation; SD, standard deviation. ^a^*P* < .001; ^b^*P* < .01; ^c^*P* < .05.

 Further, when compared to the stringency of the measures, the number of daily hospitalizations yielded a less strong and pronounced pattern of correlates with the various outcomes. Number of daily hospitalizations related positively to behavioral adherence, autonomous motivation, and perceived infection. Rather surprisingly, number of daily hospitalizations was unrelated to perceived severity of infection. Finally, daily hospitalizations correlated with one of the three basic needs (ie, lower competence) and three of the four well-being outcomes, that is, people reported somewhat higher symptoms of anxiety and lower vitality and life satisfaction on days when more hospitalizations were recorded. The stringency index yielded a stronger pattern of correlates, with stringency being positively correlated with adherence, autonomous motivation, perceived severity, and symptoms of anxiety and depression and being negatively correlated with amotivation, need satisfaction, vitality, and life satisfaction.

###  Primary Analyses

 The output of the linear mixed regression models with standardized coefficients and model information can be found in Tables 2 and 3. In general, no models showed indication for multicollinearity. As a main effect, the stringency of the measures was positively related to adherence, autonomous motivation, perceived severity as well as symptoms of depression and anxiety, while being negatively related to perceived infection, satisfaction of all three psychological needs, vitality, and life satisfaction. A similar pattern of findings emerged for hospitalization load, although no unique effect was found for controlled motivation and amotivation. First, these main effects of both predictors are distinct from the effect of the crisis phase, which showed that the second phase included lower adherence, autonomous motivation, risk perception and need satisfaction, and higher controlled motivation and amotivation. Second, these effects should be interpreted with caution given that an interaction effect emerged systematically in the prediction of all thirteen outcomes. Partial eta squares indicate that these interaction effects were moderate to large (ranging between 0.02 and 0.26).

**Table 2:** Linear Mixed Regression Models With Standardized Coefficients and Effect Sizes (Part A)

	**Adherence**		**Motivation**						**Risk Perception**			
			**Autonomous ** **Motivation**		**Controlled ** **Motivation**		**Amotivation**		**Perceived ** **Infection**		**Perceived ** **Severity**	
	**β**	**η**^2^_p_	**β**	**η**^2^_p_	**β**	**η**^2^_p_	**β**	**η**^2^_p_	**β**	**η**^2^_p_	**β**	**η**^2^_p_
Between-subject level												
Age	0.18^a^	0.03	0.21^a^	0.04	-0.21^a^	0.04	-0.23^a^	0.04	-0.04	0.00	0.25^a^	0.05
Gender [female]	0.19^a^	0.03	0.14^a^	0.02	-0.03	0.00	-0.09	0.00	0.11^a^	0.01	0.14^a^	0.02
Education level	0.02	0.00	0.02	0.00	-0.03	0.00	-0.04	0.00	0.04	0.00	-0.05	0.00
Comorbidity	0.07	0.00	0.08	0.00	-0.03	0.00	-0.04	0.00	0.07	0.00	0.17^a^	0.03
Between-days level												
Phase [second]	-0.66^a^	0.50	-0.55^a^	0.29	0.36^a^	0.10	0.69^a^	0.44	-0.36^a^	0.12^a^	-0.56^a^	0.41^a^
Hospitalizations	0.17^a^	0.07	0.12^a^	0.02	-0.09	0.01	-0.06	0.02	0.47^a^	0.21	-0.01	0.00
Stringency index	0.22^a^	0.09	0.09^a^	0.01	0.19^a^	0.03	0.04	0.01	-0.30^a^	0.09	0.42^a^	0.28
Interaction^b^	0.14^a^	0.03	0.23^a^	0.09	0.39^a^	0.14	-0.31^a^	0.16	-0.20^a^	0.05	0.15^a^	0.05
Random effects												
σ_Crisis days_	0.02		0.08		0.04		0.03		0.03		0.04	
σ_Residuals_	0.71		1.23		1.03		0.74		0.62		0.71	
Model information												
Maximum VIF	1.47		1.47		1.32		1.31		1.17		1.04	
R^2^ marginal	0.07		0.07		0.05		0.07		0.02		0.14	
R^2^ conditional	0.72		0.49		0.25		0.56		0.36		0.66	

Abbreviation: VIF, variance-inflation-factor. ^a^*P* < .001. ^b^Interaction refers to the interaction between hospitalizations and stringency index.

**Table 3:** Linear Mixed Regression Models With Standardized Coefficients and Effect Sizes (Part B)

	**Basic Psychological Needs**						**Mental Health**							
	**Autonomy**		**Competence**		**Relatedness**		**Vitality**		**Life ** **Satisfaction**		**Anxiety ** **Symptoms**		**Depression ** **Symptoms**	
	**β**	**η**^2^_p_	**β**	**η**^2^_p_	**β**	**η**^2^_p_	**β**	**η**^2^_p_	**β**	**η**^2^_p_	**β**	**η**^2^_p_	**β**	**η**^2^_p_
Between-subject level														
Age	0.21^a^	0.04	0.23^a^	0.04	0.16^a^	0.02	0.20^a^	0.03	0.16^a^	0.02	-0.25^a^	0.05	-0.24^a^	0.05
Gender [female]	0.01	0.00	-0.06	0.00	0.04	0.00	-0.05	0.00	-0.03	0.00	0.13^a^	0.02	0.09	0.00
Education level	0.01	0.00	0.03	0.00	0.04	0.00	0.04	0.00	0.03	0.00	-0.02	0.00	-0.06	0.00
Comorbidity	0.00	0.00	-0.05	0.00	-0.03	0.00	-0.06	0.00	-0.06	0.00	0.08	0.00	0.09	0.00
Between-days level														
Phase [second]	-0.02	0.00	0.30^a^	0.08	0.33^a^	0.08	0.31^a^	0.12	-0.01	0.00	-0.10	0.00	0.03	0.00
Hospitalizations	-0.08^a^	0.03	-0.05	0.01	-0.11^a^	0.01	-0.20^a^	0.06	-0.32^a^	0.08	0.46^a^	0.15	0.27^a^	0.06
Stringency index	-0.35^a^	0.09	-0.38^a^	0.11	-0.20^a^	0.03	-0.45^a^	0.21	-0.13^a^	0.01	-0.09	0.01	0.35^a^	0.09
Interaction^b^	0.45^a^	0.20	0.08^a^	0.03	0.32^a^	0.11	0.09^a^	0.02	0.39^a^	0.14	-0.32^a^	0.10	-0.30^a^	0.09
Random effects														
σ_Crisis days_	0.14		0.05		0.04		0.02		0.03		0.02		0.01	
σ_Residuals_	2.64		2.32		2.09		1.03		1.04		0.62		0.44	
Model information														
Maximum VIF	1.46		1.47		1.47		1.24		1.48		1.47		1.49	
R^2^ marginal	0.04		0.05		0.02		0.04		0.03		0.07		0.06	
R^2^ conditional	0.28		0.36		0.3		0.53		0.22		0.25		0.26	

Abbreviation: VIF, variance-inflation-factor. ^a^*P* < .001. ^b^Interaction refers to the interaction between hospitalizations and stringency index.

**Figure 2:**
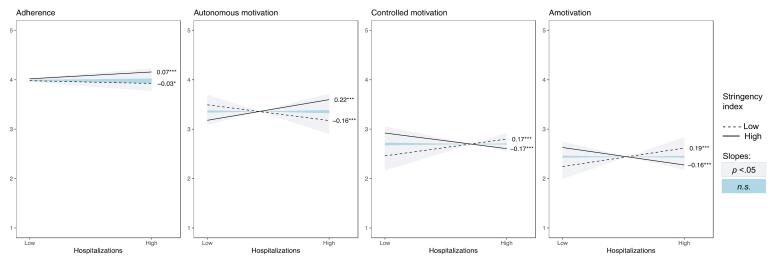
Visualizations of Interaction Effects for Adherence and Types of Motivation With Standardized Simple Slope Coefficients. *Note*: * *P* <.05, ** *P* <.01, *** *P* <.001; ‘Low’ and ‘High’ refer to the standard deviations, respectively, under and above the mean of the variable. n.s. refers to the values of the stringency index (as moderator) for which the association between hospitalization and the outcome is not significant, from left to right: [-0.94, -0.14], [-0.29, -0.00], [-0.08, 0.15], and [-0.07, 0.13].

**Figure 3:**
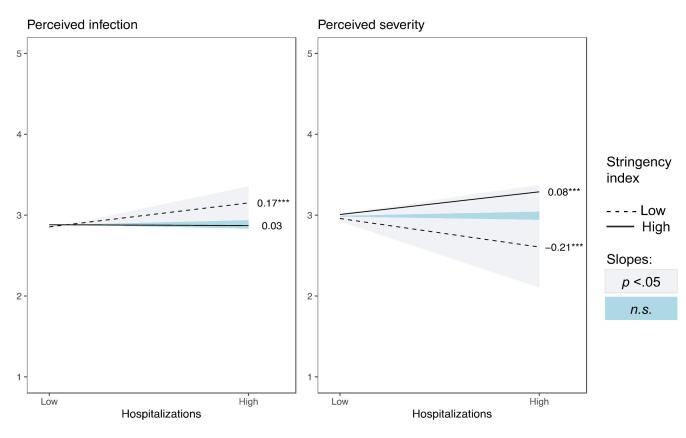
Visualizations of Interaction Effects for Risk Perception With Standardized Simple Slope Coefficients. *Note*: * *P* <.05, ** *P* <.01, *** *P* <.001; ‘Low’ and ‘High’ refer to the standard deviations, respectively, under and above the mean of the variable. n.s. refers to the values of the stringency index (as moderator) for which the association between hospitalization and the outcome is not significant, from left to right: [0.53, 1.25] and [-0.01, 0.28].

**Figure 4:**
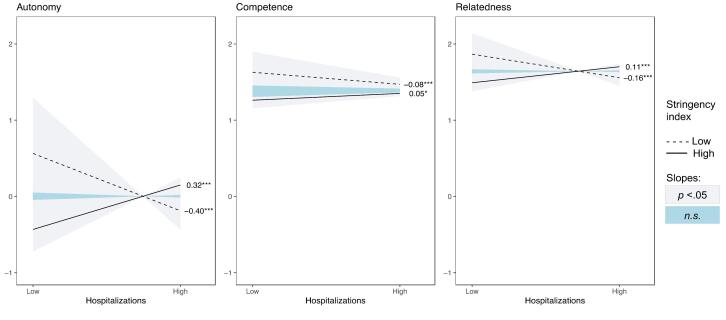
Visualizations of Interaction Effects for Basic Psychological Needs With Standardized Simple Slope Coefficients. *Note*: * *P* <.05, ** *P* <.01, *** *P* <.001. We only showed the y-axis from -1.5 to 2.5, instead of the full -4 to +4 range, for the sake of interpretability; ‘Low’ and ‘High’ refer to the standard deviations, respectively, under and above the mean of the variable. n.s. refers to the values of the stringency index (as moderator) for which the association between hospitalization and the outcome is not significant, from left to right: [0.03, 0.22], [-0.05, 0.76], and [0.06, 0.34].

**Figure 5:**
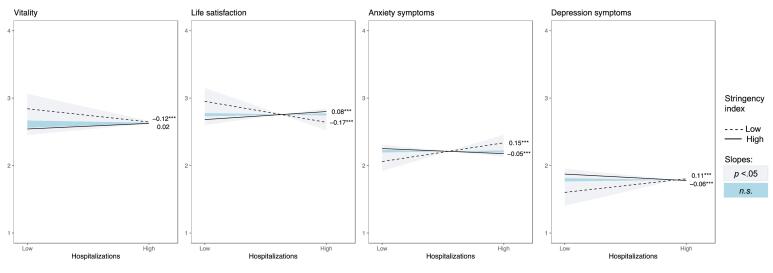
Visualizations of Interaction Effects for Well-Being With Standardized Simple Slope Coefficients. *Note*: * *P* <.05, ** *P* <.01, *** *P* <.001; ‘Low’and ‘High’ refer to the standard deviations, respectively, under and above the mean of the variable. n.s. refers to the values of the stringency index (as moderator) for which the association between hospitalization and the outcome is not significant, from left to right: [0.17, 0.87], [0.29, 0.64], [0.41, 0.78], and [0.21, 0.54].

 The pattern of interactions was similar for all outcomes, with the effect of high versus low hospitalization load being reduced to non-significance or even reversed as a function of the stringency of the measures. Whereas under conditions of high stringency, high versus low hospitalization load contributed positively to adherence, autonomous motivation (Figure 2), perceived severity (Figure 3), all three need satisfactions (Figure 4), life satisfaction, and symptoms of anxiety and depression (Figure 5), a negative association emerged under conditions of low stringency. When observing the Johnson-Neyman intervals, perceived infection is the only variable with only significant slopes for low values, indicating that higher hospitalizations never significantly resulted in lower perceived infection.

**Figure 6:**
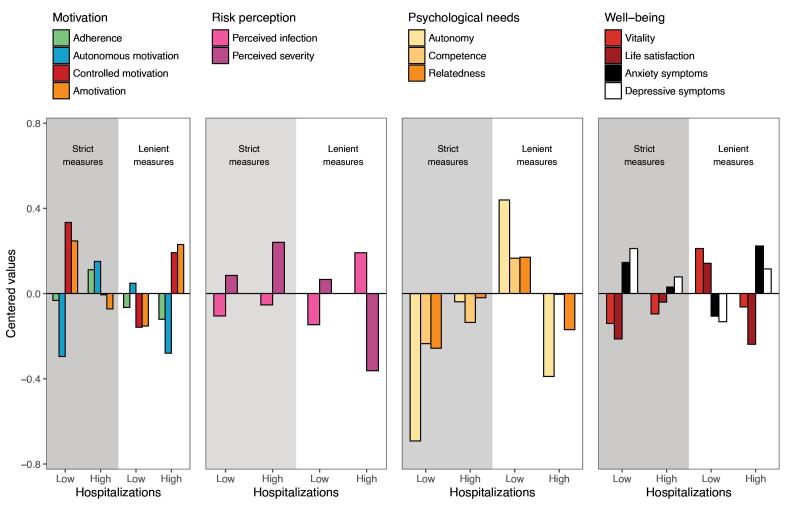
Summarized Figure of Interactions for the *High* and *Low* Levels of Hospitalizations and Stringency Index. Note. The bars in this figure refers to the values of low and high hospitalization numbers and stringent measures, based on the interaction effect visualized in Figures 3-5.

 Although the interaction analyses allow one to examine whether the contribution of hospitalization load differed as a function of stringency, Figure 6 provides a more detailed insight through direct mean-level comparison of the four critical cells in the interaction figures. For the sake of interpretability, the proportional situations (strict-high, lenient-low) are visualized as the two sets of bars in the middle of each panel. Congruent with our reasoning, we were especially interested in contrasting (*a*) high versus low hospitalization load in case of strict measures (ie, first two columns for each outcome; grey zone in Figure 6) and (*b*) high versus low hospitalization load in case of lenient measures (ie, last two columns for each outcome, white zone in Figure 6).

 When hospitalizations were high compared to low, strict measures came with more adherence and autonomous motivation, less controlled motivation and amotivation, a higher perceived risk for infection and severity, less psychological need frustration, more vitality and life satisfaction, and less anxiety and depressive symptoms. In case of lenient measures, the opposite pattern emerged: high relative to low hospitalizations resulted in less adherence, less autonomous motivation, more controlled motivation and amotivation, more perceived infection and less perceived severity, more need frustration, less vitality and life satisfaction, and more symptoms of anxiety and depression.

## Discussion

 During the COVID-19 pandemic, governments had to navigate between on the one hand controlling the epidemiological situation by imposing behavioral restrictions and on the other hand maintaining people’s motivation to adhere to the measures, enforcing the mental health of the population and avoid societal rebelliousness.^43,44^ Duringpost-pandemic times, different countries took the initiative to install committees to reflect on and evaluate the management of the COVID-19 crisis and what could be learned for future crises. The present study, that is based on a large dataset collected throughout the entire pandemic, is of direct added value to such evaluations as we examined the interplay between the epidemiological situation (based on the actual daily hospitalizations) and the stringency of the measures (based on the stringency index) in the prediction of people’s psychological and behavioral functioning. Assessing a wide range of critical outcomes throughout the pandemic, including people’s adherence, motivation, risk perception and mental health, we predicted and indeed found an intriguing pattern of findings.

###  The Role of Proportional Stringency of Health-Protective Measures

 Day-to-day variation in hospitalization load and stringency predicted uniquely day-to-day variation in all outcomes. Yet, the most striking and consistent pattern of findings concerns the hypothesized two-way interaction effects. The effect sizes of these interactions were in many cases the highest and their interplay *qualified *the observed main effects for hospitalization load and stringency. Our findings highlight the importance of approaching the psychological effects of the pandemic through the perspective of proportionality (or fit), rather than solely through the main effects of both. A comparison of the mean level differences of the four extreme cells (ie, high-low, strict-lenient) in Figure 6 provides a more detailed insight. Overall, the best outcomes are obtained when the stringency of the measures were proportional to the urgency of the epidemiological situation, as indexed by hospitalization load. In line with the proportionality hypothesis, strict measures come with more favorable outcomes in case of high hospitalization load, while lenient measures come with more favorable outcomes in case of low hospitalization load.

 On days when measures were disproportionally strict (ie, low-strict; cell 1 in Figure 6), people’s well-being was compromised, with citizens reporting more symptoms of anxiety and depression and lower life satisfaction and vitality. A variety of factors could potentially play a role herein. First, on such days, the basic psychological needs for autonomy, competence, and relatedness may be under threat, thus entailing experiences of need frustration which is a robust predictor of madadjustement.^45^ While people may accept the restriction of freedom, this is no longer the case when the stringency of the measures is disproportional to the hospitalization load. Second, the elevated anxiety may reflect citizens’ concerns regarding how long these strict measures would last and whether they would ever get removed, raising worries regarding the predictability of the situation. Third, people may begin to look back at the time when no strict measures were imposed on them. Such a comparison may elicit feelings of resentment, if not anger and even rebelliousness, which explains why their controlled motivation and amotivation to adhere to measures is highest under these circumstances.^46^ Hence, to preserve people’s motivation, need satisfaction and mental health, strict measures can better be withdrawn as soon the epidemiological situation allows.

 In contrast, when strict measures were proportional (ie, high-strict; cell 2 in Figure 6), a more adaptive pattern appears. In spite of the presence of strict measures, citizens report higher adherence and display a more adaptive motivational pattern characterized by high autonomous and low controlled motivation and low amotivation. Presumably the risk to become severely ill makes strict measures perceived as a proportional and, therefore, internalized.^47,48^ Internalized measures are considered adequate responses to handle the health threat and result in more autonomously motivated compliance.^46,49^ Also in terms of need satisfaction, strict measures do not come by definition with a loss of need-satisfying opportunities. After all, strict measures were often taken on moments when the situation was highly uncertain and, hence, strict measures would potentially bring back a sense of control and safety in life. In other words, strict measures may contribute to preserving people’s well-being and serving as a buffer against a potential rise in anxiety. Prior work in other contexts such as schooling^50^ and procedural justice^26^ similarly suggests that measures and rules are less likely to thwart people’s basic psychological needs and well-being when they are perceived to be proportional.^25^

 Much as strict measures are not inherently ‘bad’ or ‘good,’ this is also the case for lenient measures: also lenient measures can be disproportional in relation to the epidemiological situation (ie, lenient-high; cell 4 in Figure 6). On such days, people report more maladjustment compared to a situation characterized by a proportional lenient policy (ie, lenient-low). When a government fails to take action when the hospitalization load is high, people report higher anxiety and depressive complaints, lower need satisfaction, and they display a maladaptive motivational pattern. The anxiety arising under these circumstance may now be due to people’s health concerns, induced by the absence of a swift and coordinated action to prevent a further escalation of the situation. In light of governments’ failure or courage to introduce strict measures, people seem to become demotivated. They question the overall strategy of the government and may no longer believe that their actions result in desirable outcomes.

 Three additional findings deserve being mentioned. First, across the four situations, the most optimal situation is when lenient measures are proportional. ie, lenient-low; cell 3 in Figure 6. Such days are marked by the lowest levels of controlled motivation and amotivation, low perceived infection, the highest levels of need satisfaction, the highest well-being (ie, vitality and life satisfaction) and the lowest ill-being (ie, anxiety and depressive symptoms). One could approach such period as a crisis-absent situation, as neither measures nor hospitalizations are high on such days.

 Second, both types of risk perception — the probability and severity component — show a different pattern. The highest levels of perceived infection rate are reported on days with disproportional lenient measures, while perceived severity is the lowest on such days. Although people perceived a high risk to be infected, the low stringency of the measures apparently results in a low perceived severity of symptoms. Apparently, this suggests that the stringency of the measures qualifies the meaning of the epidemiological situation. In line with this reasoning, we note that the perceived severity only follows the hospitalization numbers on days with proportionally strict measures.

 Third, we found significant differences for the phase of the crisis. Our dataset includes data collected across two years of the COVID-19 crisis in Belgium, but obviously, across time several fundamental parameters changed. For instance, the vaccination campaign starting from March 2021 for the general population and the rising of COVID-19 variants with different features (eg, Omicron) should be considered, as they affected people’s perception to be infected and to have severe symptoms, and their motivation to adhere health-protective measures.^51^ As another example, some authors addressed the concept of ‘pandemic fatigue’ as the perceived inability to keep up with the restrictions.^52^ The current findings support this idea, with the second part of the crisis having significantly lower levels of behavioral adherence and autonomous motivation, higher controlled motivation and amotivation, lower risk perception, more need frustration and lower vitality. However, effect sizes differed. Although it could be expected the pandemic impacted people’s mental health significantly, it was especially for these variables that the lowest effect sizes were found.

###  Implications for Policy and Future Crises

 The current findings are of utmost importance for policy-makers as they provide a unique and informative insight in the effects of the conducted policy on diverse aspects of people’s psychological functioning. A critical question is how disproportional situations could have been avoided by policy-makers. In our view, the introduction of a “corona barometer” is critical.^53^ A corona barometer is a color-coded schema in which each color represents a set of measures that become operational in accordance with predefined epidemiological thresholds (eg, hospitalization numbers, for examples, see Ireland and New-Zealand). This entails a number of psychological advantages, including a greater sense of control and predictability for both policy-makers and citizens and may help to install a balanced or proportional set of measures in accordance with the epidemiological situation. A corona barometer would also allow the population to better anticipate upcoming political decisions in view of the changing epidemiological situation and support policy-makers to communicate clearly.^54^ This may help people not only to prepare better for new restrictions but also to take greater responsibility for their behavior in the actual epidemiological context and to induce a sense of “ownership” of the measures. Due to the lack of a colour-coded schema in Belgium, people were often surprised by unexpected (in time) and disproportional (in stringency) political interventions, which were perceived to be based on unclear and undefined criteria.

 Our findings may also relate to the role of perceived legitimacy and procedural justice.^25^ Procedural justice refers to the public perception that authorities’ decisions are fair and justifiable, resulting in more positive feelings,^55^ higher trust,^56^ and more autonomous motivation.^26^ Even when politicians have to take tough decisions, the principles of procedural justice may work as an important moderator for their psychological effects. That is, even when measures are intrusive and demanding on the part of the citizens, communication that is open, transparent, timely, and informed should buffer for its negative impact. By cultivating this notion of proportionality, politicians might not only enhance the legitimacy of their actions, but also the perception of them as taking care of the concerns of the population with both competence and benevolence.^57^

###  Limitations

 The current study involved the collection of multiple cross-sectional waves as independent groups of participants took part in the study across time. Due to the lack of longitudinal data across time, we are only able to compare mean-level differences between days to shed light on the direction of effects. For instance, although the slow introduction of strict measures in times of increasing hospitalization numbers may have caused growing levels of anxiety, anxiety may also have prevented policy-makers from introducing stringent measures which they feared would deteriorate individuals’ well-being.

 The sample itself was rather self-selective as only individuals with internet access and both the understanding and willingness to complete a questionnaire participated in the study. Also in terms of sociodemographical variables, we had a higher prevalence of women having a partner and a higher education. As previous research already demonstrated the significant role of these factors in the current study variables, with especially male, being single and having a lower education resulting in lower autonomous motivation, lower well-being and lower adherence, the absolute means in terms of the population might be underrepresented in the current findings. This is the reason why we especially focused on the structural associations within the current dataset.

 The present proportionality hypothesis draws upon the idea that measures vary in their level of perceived legitimacy.^58^ Yet, this underlying mechanism was not tested as such, as has been the case in earlier research.^49^ Future research may more directly test the mediational role of this psychological mechanism to account for the interplay between stringency and hospitalization load on people’s motivation, risk perception, and well-being. It is important to consider the actual meaning of a (dis)proportional situation. For the sake of interpretation, we currently displayed the predicted values for ‘Low’ and ‘High’ values, corresponding one SD from the mean of the stringency index and (logged) hospitalization numbers. Of course, whether the numbers absolutely represent lenient or strict measures or low or high hospitalizations remains open to debate. These labels were currently determined based on a data-driven approach. Admittedly, and although the literature lacks well-established recommendations about these issues, a epidemiological perspective on these results might provide a different interpretation of these results.

## Conclusion

 Given the uncertain character of the COVID-19 pandemic, national authorities faced the challenge to react appropriately as the epidemiological situation evolved. In the current study, we examined on a daily level how the interplay between the actual epidemiological situation and the objectively reported stringency of the measures affected people’s self-reported adherence, motivation, risk perception, need satisfaction, and well-being. Results showed that when the governmental interventions were not proportionate to the epidemiological situation, lower levels of adherence, autonomous motivation, need satisfaction, and well-being ensued. Specifically, when lenient measures were disproportional, respondents reported even more controlled motivation, amotivation and risk of infection. These results are striking, as they provide a hitherto unsuspected view on how health-protective measures may shape the effects of the pandemic on people’s behavioral and psychological functioning.

## Acknowledgements

 The Motivation Barometer is an initiative launched by the University of Ghent, and it eventually brought together researchers from the University of Leuven, the Université catholique de Louvain, and the Université libre de Bruxelles. The Motivation Barometer was continued throughout the pandemic thanks to funding provided by the University of Ghent and the Belgian Ministry of Public Health.

## Ethical issues

 The project was approved by the ethical committee of Ghent University, Belgium (N° 2020/37). Informed consent was obtained from all the participants. All methods/protocols were performed in accordance with the relevant guidelines and regulations.

## Competing interests

 Authors declare that they have no competing interests.

## Data Availability Statement

 All analysis code, and research materials are available at Open Science Framework (https://osf.io/sa498/). Correspondence concerning this article should be addressed to Joachim Waterschoot, Faculty of Psychology, Department of Developmental, Personality, and Social Psychology, Ghent, Belgium. Email: Joachim.Waterschoot@ugent.be, Tel. + 09 264 62 70.

## Funding

 This work was supported by the Belgian Federal Ministry of Health through RIZIV (Rijksinstituut voor ziekte- en invaliditeitsverzekering)/INAMI (institut national de maladie-invalidité). They had no role in any part of the work.

## Supplementary files

Supplementary file 1 contains Figures S1-S3.
