# Supplementary material for: How the Stringency of the COVID-19 Restrictions Influences Motivation for Adherence and Well-Being: The Critical Role of Proportionality
Source: Int J Health Policy Manag. 2023 Oct 10;12:8021. doi: 10.34172/ijhpm.2023.8021 (PMC10699813; doi:10.34172/ijhpm.2023.8021)
Supplement: Supplementary file 1 — contains Figures S1-S3. [file ijhpm-12-8021-s001.pdf]

**Article title:** How the Stringency of the COVID-19 Restrictions Influences Motivation for Adherence and Well-Being: The Critical Role of Proportionality

**Journal name:** International Journal of Health Policy and Management (IJHPM)

**Authors' information:** Joachim Waterschoot[1]*, Sofie Morbée[1], Omer Van den Bergh[2], Vincent Yzerbyt[3], Eveline Raemdonck[4], Marie Brisbois[3], Mathias Schmitz[3], Olivier Klein[4], Olivier Luminet[3,5], Pascaline Van Oost[3], Maarten Vansteenkiste[1]

[1]Department of Developmental, Personality and Social Psychology, Ghent University, Ghent, Belgium.

[2]Health Psychology, Faculty of Psychology and Educational Sciences, University of Leuven, Leuven, Belgium.

[3]Institute for Research in the Psychological Sciences, Université Catholique de Louvain, Louvain-la-Neuve, Belgium.

[4]Faculty of Psychological Sciences and Education, Université libre de Bruxelles, Bruxelles, Belgium.

[5]Fund for Scientific Research (FRS-FNRS), Brussels, Belgium.

*__Correspondence to:__ Joachim Waterschoot; Email: Joachim.Waterschoot@ugent.be

**Citation:** Waterschoot J, Morbée S, Van den Bergh O, et al. How the stringency of the COVID-19 restrictions influences motivation for adherence and well-being: the critical role of proportionality. Int J Health Policy Manag. 2023;12:8021. doi:10.34172/ijhpm.2023.8021

**Supplementary file 1.**

*Figure S1*. Daily total of hospitalized COVID-19 patients in Belgium

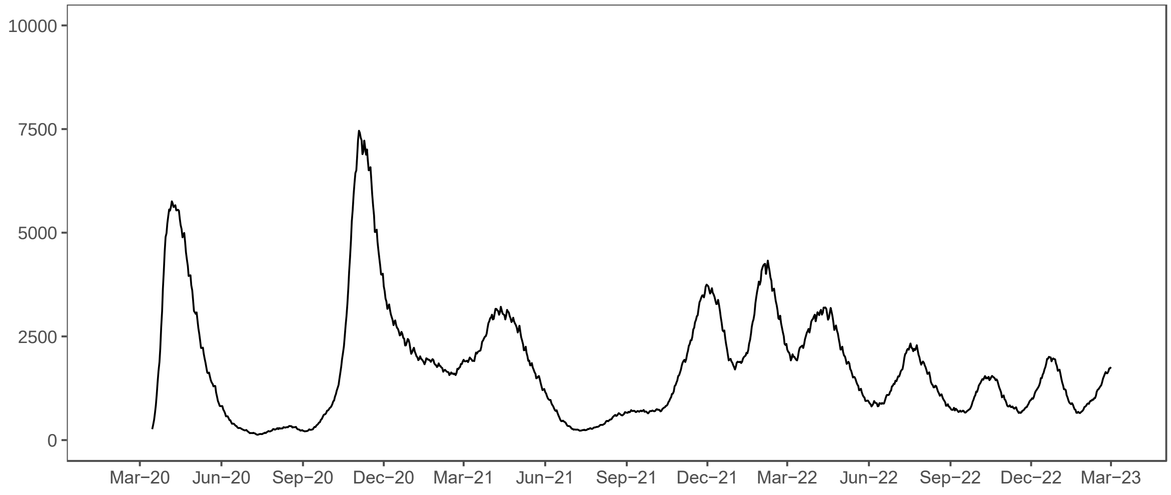

*Figure S2.* The COVID-19 Stringency index in Belgium

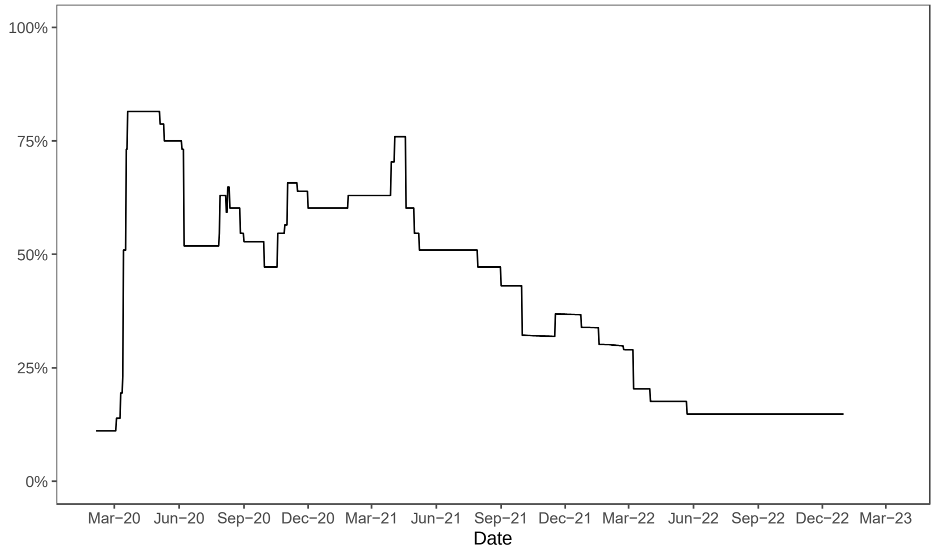

*Figure S3.* Visualization of Johnson-Neyman interval in prediction of adherence

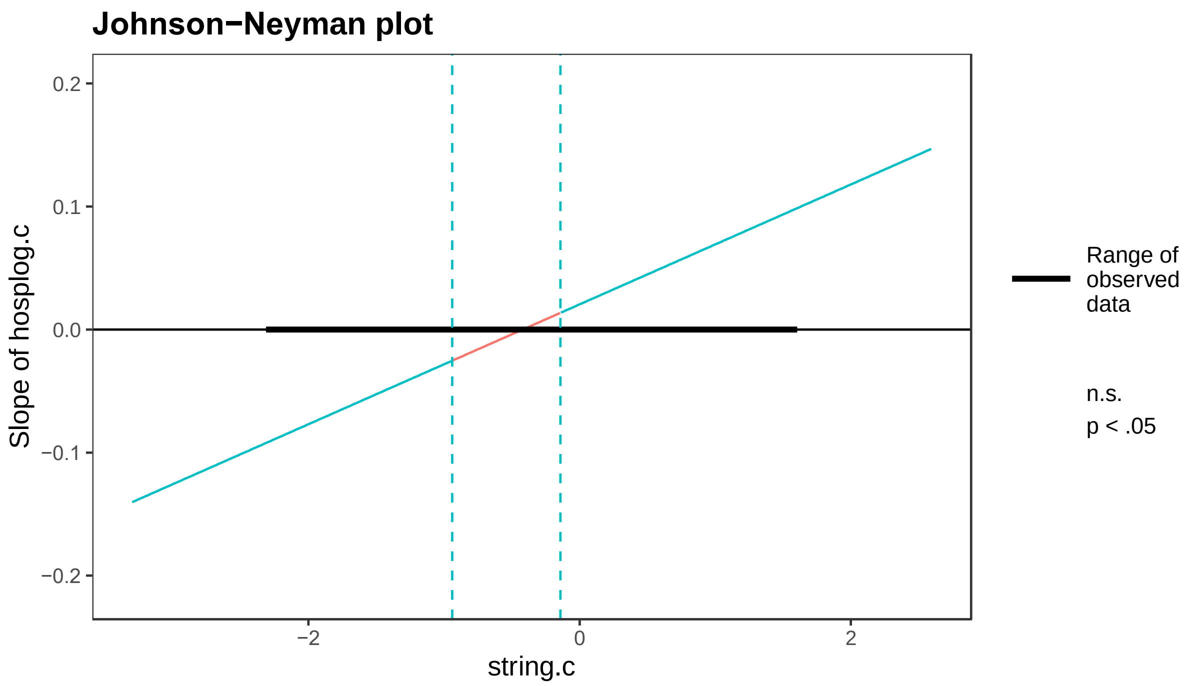


*Note. String.c = (standardized) stringency index; hosplog.c = (standardized and logged) hospitalization numbers; the blue line refers to the hospitalization-stringency association in prediction of the current outcomes 'adherence' across values of the moderator 'stringency index'. Herein, it is showed that this association is not significant in the moderator values -0.94 to -0.14 (red part of the line). The blue shadow refers to the 95%-confidence interval.*
